# Understanding context of violence against healthcare through citizen science and evaluating the effectiveness of a co-designed code of conduct and of a tailored de-escalation of violence training in Eastern Democratic Republic of Congo and Iraq: a study protocol for a stepped wedge randomized controlled trial

**DOI:** 10.1186/s13063-023-07839-3

**Published:** 2023-12-19

**Authors:** Giovanfrancesco Ferrari, Samuel Makali Lwamushi, Ghislain Bisimwa Balaluka, Riyadh K. Lafta, Christian Schindler, Daniella Bugugu, Emmanuel Lurhangire, Fabrizio Tediosi, Jessica Ramirez Mendoza, Sonja Merten

**Affiliations:** 1 Swiss Tropical and Public Health Institute, Kreuzstrasse 2, 4123 Allschwil, Switzerland; 2 Universität Basel, Petersplatz, 1, P.O. Box, CH-4001 Basel, Switzerland; 3 Ecole Régionale de Santé Publique, Université Catholique de Bukavu (ERSP-UCB), Bukavu, Democratic Republic of the Congo; 4 College of Medicine, Al Mustansiriyah University, Baghdad, Iraq; 5 International Committee of the Red Cross, Geneva, Switzerland

**Keywords:** De-escalation of violence training, Violence, Health care worker, Citizen science, Code of conduct

## Abstract

**Background:**

Violence against health care workers (HCWs) is a multifaceted issue entwined with broader social, cultural, and economic contexts. While it is a global phenomenon, in crisis settings, HCWs are exposed to exceptionally high rates of violence. We hypothesize that the implementation of a training on de-escalation of violence and of a code of conduct informed through participatory citizen science research would reduce the incidence and severity of episodes of violence in primary healthcare settings of rural Democratic Republic of Congo (DRC) and large hospitals in Baghdad, Iraq.

**Methods:**

In an initial formative research phase, the study will use a transdisciplinary citizen science approach to inform the re-adaptation of a violence de-escalation training for HCWs and the content of a code of conduct for both HCWs and clients. Qualitative and citizen science methods will explore motivations, causes, and contributing factors that lead to violence against HCWs. Preliminary findings will inform participatory meetings aimed at co-developing local rules of conduct through in-depth discussion and input from various stakeholders, followed by a validation and legitimization process. The effectiveness of the two interventions will be evaluated through a stepped-wedge randomized-cluster trial (SW-RCT) design with 11 arms, measuring the frequency and severity of violence, as well as secondary outcomes such as post-traumatic stress disorder (PTSD), job burnout, empathy, or HCWs’ quality of life at various points in time, alongside a cost-effectiveness study comparing the two strategies.

**Discussion:**

Violence against HCWs is a global issue, and it can be particularly severe in humanitarian contexts. However, there is limited evidence on effective and affordable approaches to address this problem. Understanding the context of community distrust and motivation for violence against HCWs will be critical for developing effective, tailored, and culturally appropriate responses, including a training on violence de-escalation and a community behavioral change approach to increase public trust in HCWs. This study aims therefore to compare the effectiveness and cost-effectiveness of different interventions to reduce violence against HCWs in two post-crisis settings, providing valuable evidence for future efforts to address this issue.

**Trial registration:**

ClinicalTrial.gov Identifier NCT05419687. Prospectively registered on June 15, 2022.

**Supplementary Information:**

The online version contains supplementary material available at 10.1186/s13063-023-07839-3.

## Introduction

### Background and rationale {6a}

Violence against health care workers (HCWs) and alarming levels of distrust towards the medical profession are global public health emergencies especially in fragile and conflict-affected settings [1–4]. Research on interventions addressing violence against HCWs rarely focuses on conflict (5.0%), post-conflict (1.5%), or fragile setting (1.9%) [5]. There is emerging evidence that individual educational interventions including violence de-escalation techniques for HCWs are effective in reducing aggression or reducing the severity of such episodes [6, 7]. Increased knowledge, skills, and self-confidence in managing aggressions towards staff have been reported as potential benefit of de-escalation interventions among HCWs [5, 8–10]. Evidence also suggests that when these interventions are combined with contextual interventions addressing cultural aspects or organizational practices and policies at various levels of the health system, including security measures, they are more likely to be effective [5, 7, 8, 11, 12]. Several studies further showed a significant reduction in lost workdays, improved staff retention, and reduced overall expenditure among mental health professionals who received a violence de-escalation training [13]. However, systematic literature reviews highlighted weaknesses in the design of many studies, such as low statistical power or the absence of control groups [7, 8]. Further, there is limited understanding of the broader economic implications of reducing violence against HCWs and the costs of implementing interventions, particularly in fragile settings where violence against HCWs is prevalent [1, 2, 5]. In Iraq, more than 85% of HCWs in the city of Baghdad reported high levels of exposure to violence associated with dissatisfaction of care, particularly from patient’s relatives [14]. Medical doctors are being assaulted, threatened, abducted, and humiliated, which prompts many of them to leave the country [14–16]. The eastern provinces of the Democratic Republic of Congo (DRC) have been affected by decades of instability and HCWs face similar challenges, with a very recent history of attacks by the local population [17–19].

To address the issue of violence against healthcare, the International Red Cross and Red Crescent Movement launched the Health Care in Danger (HCiD) initiative in 2011 and developed preventive measures to ensure the safety and security of health personnel including a de-escalation violence training to equip HCWs with communication skills to de-escalate verbal and physical aggressions by patients and their relatives [20, 21]. Furthermore, efforts to prevent posttraumatic stress among HCWs victims of violence have been resurfacing [22, 23]. The de-escalation violence training was previously evaluated in a large hospital in Pakistan, where it was found to enable HCWs to better respond to aggressive behavior, preventing an escalation of violence and reducing posttraumatic stress [22]. The evaluation, however, identified a need to intervene, in addition, at the organizational and contextual level, to further reduce the incidence of violence against HCWs. The relevance of organizational and contextual interventions had previously been highlighted in other contexts as well, but evidence on the effectiveness of these interventions is limited [5, 6, 24].

Thus, to address the evidence gap on intervention effectiveness, this study proposes to complement the de-escalation of violence training developed with a context-specific organizational level intervention in the form of a code of conduct co-designed by HCWs and the local communities, based on a formative citizen science research [25–28]. The citizen science and co-design approach will allow for the investigation of contextual antecedents, motivations, and causes of violence to tailor the interventions in vulnerable settings, while also ensuring community participation and increasing community trust in HCWs. The implementation of the code of conduct will involve additional government stakeholders to ensure that the code of conduct is enforced effectively [29]. With a stepped-wedge design, this study will provide robust evidence on the effectiveness, cost, and consequences of addressing violence against HCWs in two fragile contexts affected by alarming levels of violence, the Eastern South Kivu province in the DRC, and Baghdad, Iraq, as well as scalability and transferability.

### Objectives {7}

The project's overarching goal is to determine whether a training on de-escalation of violence for HCWs, and a publicly displayed Code of Conduct for both HCWs and clients at the health facilities, can reduce the incidence and severity of violent episodes, and to identify the most cost-effective way to implement interventions in rural and urban areas of low– and middle-income countries (LMICs).

**Fig. 1:**
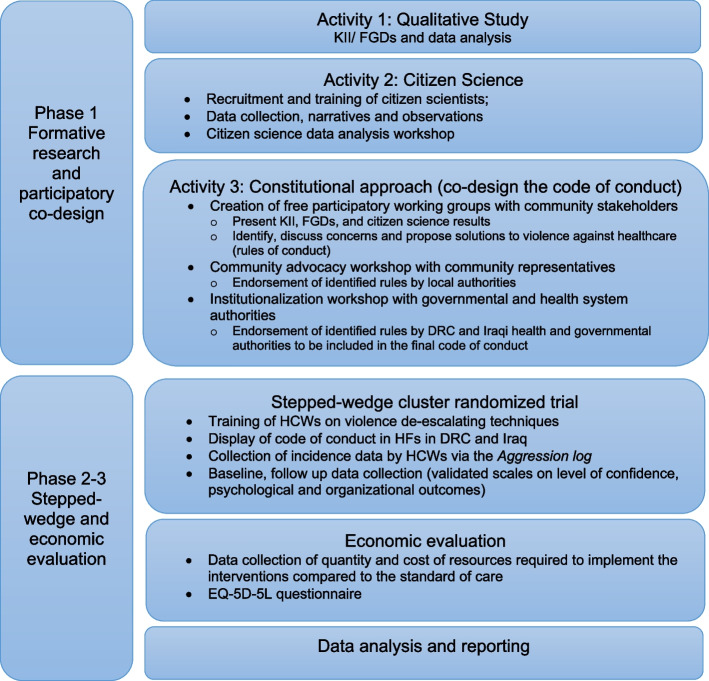
Workflow and design methodology

The study will include three phases to meet this objective. In phase 1, qualitative research methods such as citizen science and other community participatory methods will be used to investigate the triggers, causes, and motivation behind violence. The aim is to propose, co-design, and validate a set of rules of conduct to be implemented at the level of health facilities in rural DRC and Baghdad. Phase 2 will be a stepped-wedge randomized-cluster trial (SW-RCT) that evaluates the effectiveness of the de-escalation violence training in conjunction with the code of conduct. Finally, in phase 3, the financial and economic cost of implementing the interventions will be estimated, along with its cost-effectiveness compared to no intervention. A schematic representation of the research project design and workflow is presented in Fig. 1.

## Phase 1: Formative research and participatory intervention co-design phase

### Primary objective

In phase 1, the study will involve the community in all aspects of the research through citizen science and participatory methods. By doing so, the study aims to gain a deeper understanding of the motivations, causes, and contributing factors that lead to violence against HCWs. This understanding will inform the development of a culturally appropriate code of conduct and the adaptation of the de-escalation of violence training.

### Secondary objectives

To achieve the primary objective, the study phase 1 pursuits the following secondary objectives:

- To identify, triggers, causes, and types of reactive violence against HCWs along with the characteristics of their perpetrators in two different post-crisis settings;
- To understand underlying motivations of violence against HCWs;
- To determine the profile characteristics of HCWs that are most likely exposed to violence;
- To identify the profile characteristics of the victims of violence with a focus on differences in violence exposure between HCWs occupation categories and gender differences;
- To adapt and implement an existing training of de-escalation of violence for HCWs tailored to the causes of violence identified (individual-level intervention);
- To develop and validate a set of rules that can be implemented at the level of the health facility, such as a code of conduct, through citizen science and other participatory methods (structural-level intervention);

### Primary and secondary endpoints

Qualitative endpoint of study phase 1 (formative research and participatory intervention co-design phase )

- Co-designed case scenarios for the de-escalation of violence training;
- Co-designed culturally-appropriate rules at the health facility level to prevent violence and reduce the distrust towards HCWs and the health system;

## Design of the formative research and participatory co-design phase

Phase 1 will use a transdisciplinary citizen science approach to adapt the de-escalation of violence training intervention and the code of conduct to reduce violence towards HCWs. The research will involve a qualitative study, citizen science, and a constitutionality approach to develop culturally appropriate rules [29, 30]. The results of the study will be used to implement warning boards of conduct and adapt the de-escalation of violence training to include real case scenarios. The scenarios will help HCWs to improve communication, decision-making, and medical ethical considerations in stressful situations.

### Activity 1: Qualitative study

#### Instruments and study population

The aim of the qualitative study is to gain first-hand insights into the community's perceptions of violence against HCWs through structured dialogues and active participation from identified stakeholders. Community members for interviews will be selected via community mapping exercises and consultations with local leaders and HCWs, ensuring active involvement and diverse representation. The qualitative study will be conducted using key informants interviews (KII), with a purposive sample of policy makers, practitioners, health facility managers and HCWs, and focus group discussions (FGDs) with community members and groups of concern beneficiaries of health care services. Each focus group will be composed of 6–8 purposively selected participants.

#### Data collection

Interview guides will be developed to gather information on knowledge, perceptions, causes, and triggers of violence against HCWs in the community, perpetrators of violence and reasons for verbal/nonverbal violence compromising the delivery, quality, and accessibility of health care. Protective factors and stakeholder experiences with violence will also be investigated. Interviews and FGDs will be conducted by a trained social scientist and a note-taker in a suitable community location, recorded with consent, and later on be transcribed and translated and stored on a secure server in Basel, Switzerland, with anonymized data.

Recognizing the potential trauma among HCWs due to past violence, the study will prioritize participant well-being by employing informed consent, empathetic interviews, and continuous psychological support, fostering a safe and supportive environment for emotional well-being.

#### Data analysis

The narratives from focus-group discussions will be analyzed using framework analysis with the help of qualitative data analysis software (Atlas TI) [31]. Additional themes will be identified by comparing codes and re-reading of the entire interviews. The local research team will conduct the analysis. Each country PI will lead the qualitative study.

### Activity 2: Citizen science

As part of the formative research phase, the citizen science component will aim to complement and deepen the understanding of the research question and of the data collected through the qualitative interviews and FGDs. Citizen science will use the unique view of community members who will serve as peer-interviewers of their fellow community members to provide additional contextual information helpful in understanding the issue of violence against healthcare.

#### Instruments and measures

As part of the citizen science component, two methodological approaches will be used, (1) narrative inquiries and (2) observations.

#### Narrative inquiries

The narrative inquiry technique uses storytelling for gathering information from the participants minimizing the influence from the interviewer. This technique avoids pre-structured interviews going beyond the questions-answer type of interviews thus allowing less imposed and more “valid” information from the perspective of the participant [32, 33]. Narrative inquires will thus use story-telling and listening to collect accounts of episodes of violence at both the level of the community and health facilities.

#### Observations

Citizen scientists (CSs) will be trained to conduct observations and report their findings in selected health facilities. This method will complement the interview methods and involve detailed observation and recording of people's behaviors and conversations in natural settings [33]. CSs will record any information regarding signs of overcrowding, long waiting times, tensions, or violence between patients/relatives and HCWs. Notes will be recorded in a field notebook and analyzed later, and a purposive sample of health facilities will be selected based on accessibility and safety.

#### Training of citizen scientists

CSs will be trained over 3 days on data collection methods including sessions on how to maintain confidentiality, developing empathy, and enhancing cultural sensitivity. Special emphasis will be placed on the ethical handling of sensitive information related to violence against HCWs. Following this training, CSs will engage in a 2-day pilot testing to refine their skills and familiarize themselves with real-life scenarios. Subsequently, a dedicated period of 5 days will be allocated for the data collection process.

#### Citizen science data analysis

During a 1-day workshop, CSs, with the assistance of qualitative researchers will work together on interpretation and presentation of findings. The objective of this workshop is to involve CSs in a simple analysis of data collected, engaging them in the discussion of individual narratives and observations and to summarize the priority issues identified. In a second phase, qualitative researchers will present the results from KII and FGDs to the CSs. This will help to identify common themes and insights regarding the perception of violence against HCWs. CSs together with qualitative researchers will summarize the overall workshop findings and will identify key-stakeholder groups to participate in the following participatory working groups.

### Activity 3: Constitutional approach

The preliminary results of the community qualitative inquiry and citizen science will be used to identify key stakeholders and form participatory working groups to discuss priority areas of intervention for preventing and responding to violence against HCWs. The aim is to generate, approve, validate, and legitimate rules of conduct at the health facility level. This process will ensure inclusiveness of all interested groups, creating a sense of ownership and allowing for local rules to be discussed with higher-level authorities during intergroup discussion. This activity will take place during three consecutive levels.

#### Creation of free participatory working groups (level 1)

This step will entail the formation of participatory working groups (PWG) without interference from the research team to discuss, share experiences, and propose solutions for violence against HCWs. This includes proposing related rules of conduct to be adopted by community members and HCWs. The research team will ensure equal representation and input from marginalized groups (women, youth, and the elderly, for example). Following the group session, a feedback day will be organized for each health facility, where the appointed person from each PWG will extract and summarize the results of the discussion to present to the community advocacy workshop (level 2).

#### Community advocacy workshop (level 2)

The community advocacy workshop (CAW) aims to validate the priorities and concerns identified during PWGs with local authorities before seeking final validation from governmental, political, and health system authorities. It will involve community and religious leaders and key stakeholders, who will be guided by the research team to identify priorities and preferred outcomes. The CAW will be conducted in both DRC and Iraq, with gender-balance ensured and a facilitator and deputy appointed to summarize the rules of conduct approved during the workshop.

#### Workshop on validation and legitimization (level 3)

**Fig. 2:**
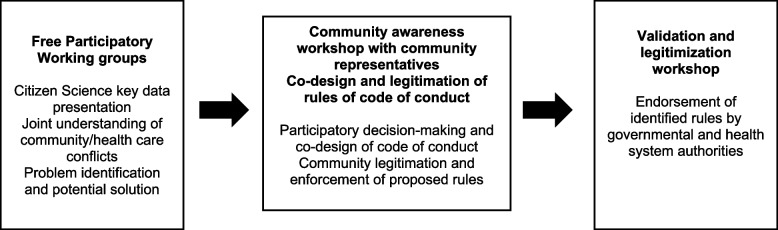
Development and institutionalization of a set of rules to be integrated in the code of conduct

A final validation and legitimization workshop will be held involving relevant government authorities (primarily the MoH, but others as needed), in order to reach a consensual written agreement for management and regulation of conflicts, as well as to validate the rules of conduct approved during the preceding steps. This phase will allow recognizing the legitimacy of the rules of conduct issuing from this process. Representatives of the humanitarian sector, including ICRC delegates, will also partake in this process (Fig. 2).

## Phase 2: Stepped-wedge cluster randomized study

### Objectives of phase 2 (stepped-wedge randomized controlled study)

Primary objectives

- To assess the effectiveness of a contextualized de-escalation training for HCWs and of a co-designed code of conduct on the incidence of physical and non-physical incidents (verbal abuse, threats, ironic language, provocative or aggressive body language, etc.) reported during the fulfilment of a professional activity in the last 6 months

Secondary objectives

Secondary objectives for study phase 2 will include assessing personal knowledge and attitudes towards aggressive behaviors and health and psychosocial consequences of violence on HCWs.

The secondary objectives of study phase 2 are thus:

- To assess the level of communication and behavioral techniques to deal with aggressive behaviors among HCWs
- To assess self-efficacy and empathy regarding aggressive behaviors among HCWs
- To assess the level of post-traumatic stress disorders among HCWs
- To assess the level of burnout among HCWs
- To assess the intention to leave among HCWs
- To assess the acceptability of the training among HCWs

## Study phase 3: Economic evaluation

### Objectives of study phase 3 (economic evaluation)

Primary objectives

- To estimate the economic cost of the intervention

Secondary objectives

- To estimate the health care services foregone or postponed;
- To estimate the medical and non-medical costs incurred by HCWs;
- To assess the number of sick leave spells taken during the study period;

### Endpoints of study phase 3 (Economic evaluation)

Primary endpoint

- Economic cost of the interventions;

Secondary endpoint

- Cost of health care services foregone or postponed due to violent episodes;
- Medical and non-medical costs incurred by HCWs

### Methods of the economic evaluation

The economic evaluation will assess the cost and the cost-effectiveness of providing the delivery of the violence de-escalation training intervention combined with the code of conduct compared with no intervention. To this end, the study will first estimate costs in different settings collecting data on resources used during the SW-RCT from the health facilities medical records. Secondly, the study will assess the incremental health benefits for HCWs following the introduction of the combined interventions to reduce violence against them [34] and the cost-effectiveness of them compare to no intervention using quality-adjusted life years (QALYs) measured through EQ-5D-5L [35, 36]. Third, the study will use standardized questionnaires to estimate medical and non-medical expenses incurred by HCWs due to violence episodes at different points in time [37]. Sensitivity analysis will be conducted to examine the uncertainty of the results, and if positive, a budget impact analysis will estimate the costs required to implement the interventions at scale in the two countries [38].

## Trial design {8}

**Fig. 3:**
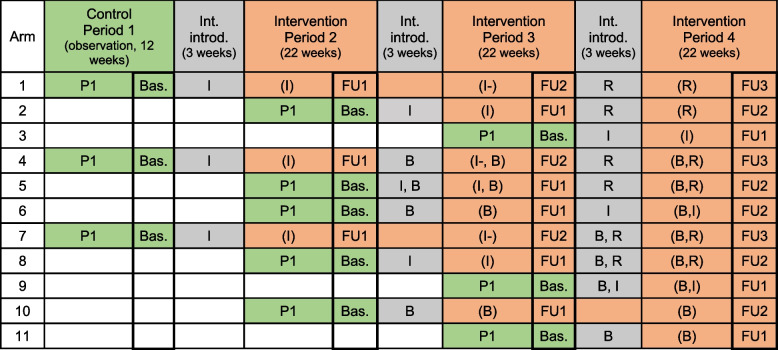
Diagram representing the stepped wedge cluster randomized design

Study phase 2 will use a stepped-wedge randomized controlled trial (SW-CRT) design, open to new individuals joining over the study duration, and with clusters transitioning from the control to the intervention arm in a random order. The trial will consist of 11 sequences with a maximum of four intervention periods, combining the de-escalation training intervention (I) followed by a refreshment training and a code of conduct board level intervention (B) with an overall study duration of 87 weeks (Fig. 3). Outcomes will be measured at four discrete points for arms of greatest length and a lower number of outcome measures for the remaining arms.

## Methods: Participants, interventions and outcomes

### Study setting {9}

A random sample of 33 rural health care facilities from within three health zones (HZs) in DRC will be selected for inclusion in the trial. In Baghdad, 22 health facilities will be chosen at random. Participants in Iraq will be recruited from the more exposed wards.

### Eligibility criteria {10}

#### Health facility inclusion criteria

- Primary level rural health facilities in DRC; urban health facilities in the city of Baghdad, Iraq
- MoH-approved health facilities

#### Health facility exclusion criteria

- Anticipated challenges with complying with the study protocol

#### Participant HCWs inclusion criteria

- Randomly selected HCWs from participating health facilities in DRC, including community health care workers (CHWs); junior doctors during their first year residence from participating health facilities in Baghdad, Iraq
- HCWs participants must have completed the written informed consent
- HCWs who have been working in the health facilities the 6 months prior to the start of the trial

#### Participant HCWs exclusion criteria

- Age < 18 years old

#### Who will take informed consent? {26a}

Individuals who agree to participate and who meet the study eligibility criteria will then be invited to an in person meeting to learn about the study and to provide written informed consent. Participants’ names will not be written on used questionnaires and schedules and no record of participants’ names will be kept. Interviews will be conducted with the strictest confidentiality at an appropriate location without any disruption in the services provision.

#### Additional consent provisions for collection and use of participant data and biological specimens {26b}

On the consent form, participants will be asked for their consent to the use of their data in the event of their withdrawal from the trial. Participants will also be requested to grant permission for the research team to share relevant data with individuals affiliated with the participating Universities or regulatory authorities, where applicable. This trial does not involve the collection or storage of any biological specimens.

## Interventions

### Explanation for the choice of comparators {6b}

The comparator will be the current standard of care, which does not include any approach to the management of aggressive or violent behavior by HCWs. The trial will compare within each cluster the incidence of violent episodes and their severity before the introduction of the de-escalation of violence training and the code of conduct with the incidence of violent episodes and their severity from health facilities, after the intervention deployment. The SW-RCT design allows each site to serve as its own control and likely reduces the potential for confounding on cluster and possibly individual-level characteristics.

### Intervention description {11a}

The intervention consists of two key components, (1) an individual capacity building component delivered through a de-escalation of violence training for HCWs and (2) a code of conduct delivered via a warning board to be implemented at the health facilities and co-developed during the formative phase. HCWs will also receive a refreshment training. The de-escalation of violence training program developed by the ICRC will be used for the implementation [39]. The training will be delivered to HCWs in participating clusters over a half-day workshop. Expert physicians from the MoH and from the research teams from the collaborating universities will co-facilitate the group sessions. The de-escalation of violence training is designed to provide HCWs with the ability to identify tense situations from the outset and to develop skills to defuse them before they further escalate. Furthermore, the training helps to improve the communication skills of HCWs with patients and their families. The training will consist of five modules: (1) an introductory module, (2) understanding violence in health-care settings: causes and effects, (3) learning key behaviors for preventing and de-escalating tense situations, (4) communicating and engaging with people, and (5) taking learning back into the workplace [39]. The training is based on behavioral theory, which states that people learn new behaviors more effectively when their needs are analyzed in a way to ensure their usefulness and applicability. The learnt behaviors provide a direct benefit to the participants when the techniques are practiced during the training. The training material will be contextualized and adapted to include real case-scenarios highlighted during the formative research phase. Once a group of clusters crosses over into the intervention phase, trainers will start the delivery of the training according to a pre-defined delivery schedule (Fig. 1). All HCWs who meet the inclusion criteria will start the training. Along with the de-escalation of violence training, a code of conduct developed during the formative phase will be introduced and displayed in the participating clusters, according to the rollout process.

### Criteria for discontinuing or modifying allocated interventions {11b}

Participants will be informed that they are free to withdraw their consent and that they can reject further participation in the trial at any time.

### Strategies to improve adherence to interventions {11c}

Each health facility will designate a contact person who will be supportive of the violence de-escalation implementation. Hospital managers will also be involved in providing their support by participating in the training and providing ongoing feedback to HCWs adhering to the intervention. Further, peer support among HCWs will be encouraged through group discussions and case presentations to help them implement the violence de-escalation intervention.

### Relevant concomitant care permitted or prohibited during the trial {11d}

The interventions are designed to change the behaviors that HCWs and clients take with regard to violence against health care. They neither require nor preclude concomitant care during the trial.

### Provisions for post-trial care {30}

Participants in the trial will be allowed to receive care if needed.

### Outcomes {12}

#### Endpoints of study phase 2 (stepped-wedge cluster randomized controlled study)

Primary endpoints

- Number of physical and non-physical incidents (verbal abuse, threats, ironic language, provocative or aggressive body language, etc.) reported during the fulfilment of a professional activity in the last 6 months;
  Secondary endpoints

Type of violence

- Number of physical and non-physical incidents reported by type, by perpetrator, by place, by time, by gender, and during the fulfilment of which activity in the last 6 months;

Coping with violence

- Level of communication skills related to workplace violence among HCWs [40];
- Clinician Confidence in Coping With Patient Aggression (CCPAI scale) [34]

Psychological endpoints

- Level of post-traumatic stress disorders (PTSD) among HCWs (PTSD Checklist for DSM-5) [41–43];
- Level of burnout among HCWs (Maslach burnout inventory scale) [44, 45];
- Psychological empathy among HCWs (Jefferson scale of physician empathy scale) [40, 46, 47];
- Job satisfaction

Organizational endpoints

- Number of sick leave spells taken during the study period;
- Intent to leave among HCWs because of violence (TIS-6 Intention to leave scale) [48, 49]

### Participant timeline {13}

#### Sample size {14}

Power simulations were performed separately for the study in Iraq and the study in DRC based on the study design scheme given in Table 1. Details of the simulations are provided in an online supplement (see Supplementary file 1). For short, the average number of events per HCWs and 6-month period was assumed to be 1 without intervention, 0.6 after intervention B, and 0.6 in the 6-month period following interventions I and R. A relapse to 0.7 was assumed for the effect of intervention I in the second period after intervention in arms 1, 4, and 7. All effects were assumed to be additive on the log-scale of expected event numbers. We further assumed average event rates to vary between facilities and between HCWs within facilities. Both variations were assumed to follow a normal distribution on the log scale of expected event numbers, with a mean value of 0 and a standard deviation of 0.2. We assumed the probability of a HCW quitting in a given period to be at most 30%. Under these assumptions, 22 health facilities in Iraq with 12 HCW per facility and 33 health facilities in DRC with 5 HCW per facility would provide more than 90% power for finding statistically significant effects of interventions B and I after pooling results from the two countries, assuming a significance level of 5%. The power to detect a significant effect of intervention R after pooling would also exceed 90% if quitting HCWs are replaced one by one.

**Table 1:** Participant timeline

			Study periods						
	Allocation		Post-allocation						Close-out
	P1 *0–12 weeks*		P2 *13–37 weeks*		P3 *38–62 weeks*		P4 *63–87 weeks*		*88–99 weeks*
Time points	0	Bas.		FU1		FU2		FU3	
Recruitment									
Eligibility screen	x								
Informed consent	x								
Demographic data	x								
Allocation	x								
Assessments									
Aggression log (continuous)	x		x		x		x		
Health care staff questionnaire		x		x		x		x	
Level of confidence (CCPAI self-efficacy)		x						x	
PTSD Checklist for DSM-5 (PCL-5)		x		x		x		x	
Level of burnout (Maslach burnout inventory scale)		x		x		x		x	
Jefferson physician empathy scale		x						x	
TIS-6 Intention to leave		x		x		x		x	
EQ-5D-5L (EuroQol 5 Dimension 5 Level) Quality of life (QoL)		x		x		x		x	
Assessment of costs		x		x		x		x	
Data analysis and reporting							x		x

Table 2 describes the expected total number of HCWs that will be recruited per country to allow for 10, 20, and 30% drop outs within the 6-months period.

**Table 2:** Results of the power simulations (500 replications). Estimated Power to detect a significant effect of the respective intervention at the 5%-level, if quitting HCWs are replaced at the beginning of the next study period

	Iraq			DRC			Summary estimate		
Probability of quitting in a given period	10%	20%	30%	10%	20%	30%	10%	20%	30%
Intervention I (individual training followed by collaborative learning)	0.98	0.98	0.97	0.94	0.90	0.89	0.99	0.99	0.99
Board intervention B	0.97	0.97	0.95	0.89	0.87	0.89	0.99	0.99	0.99
Refreshment intervention R	0.73	0.70	0.68	0.60	0.54	0.54	0.92	0.90	0.89

### Recruitment {15}

The research study team will invite potential HCWs in DRC and junior medical doctors in Baghdad from selected sites to participate in the study. A designated contact person will collaborate throughout the project to ensure the sustainability of recruitment and communication between clusters and their organizational levels. In Iraq, within each site, junior doctors will be recruited from the most exposed wards. Each HCW will be assigned a unique ID number.

## Assignment of interventions: allocation

### Sequence generation {16a}

The study statistician will perform the randomization of health facilities to receive the intervention at pre-planned steps and will establish the random order of the allocation sequence through computer-generated random numbers.

### Concealment mechanism {16b}

Only the statistician and the PI will be aware of the allocation order sequence, while the country co-investigators will be blinded to it, and only the next health facility randomized for rollout will be revealed at each crossover intervention implementation time points, approximately 4 weeks prior, to allow for training on de-escalation of violence techniques.

### Implementation {16c}

The statistician will generate the allocation sequence. HCWs from the participating sites who give consent for participation and who fulfil the inclusion criteria will be enrolled in the study.

## Assignment of interventions: Blinding

### Who will be blinded {17a}

It will not possible to blind the trial participants to the intervention, as participants will be aware of the intervention, the violence de-escalation training they are receiving. The data analyst and the outcome assessors will be blinded to the status of the intervention at the time the outcome is determined.

### Procedure for unblinding if needed {17b}

N/A. Procedures for unblinding do not apply to the trial design.

## Data collection and management

### Plans for assessment and collection of outcomes {18a}

In all arms, incidence and severity of episodes of violence will be collected by each consenting HCW (self-reported) via the *Aggression log*, longitudinally, during the initial observation period (P1) of 12 weeks and during the whole duration of the trial. This instrument will capture the frequency and description of the incident experienced during the working hours and will allow the staff to judge the severity of the incident via a 100-mm visual analogue scale (VAS). The *Aggression log*, adapted from Morken et al., was customized based on qualitative insights, and simplified for user-friendliness, incorporating feedback from healthcare personnel and experts in both countries, ensuring efficient reporting despite demanding work pressure [50]. The tool will be completed by the HCWs and each time the HCWs are exposed to aggressive behaviors, defined as any verbal, non-verbal, or physical behavior that was threatening or physical behavior that was adding arm. Entries will include time of incident, perpetrator’s gender, type of assault, whether verbal (e.g. verbal threat, insult) or physical, means used by the aggressor and consequences for the victim. Participants will be assured confidentiality and anonymity of their responses through unique identifiers, and a secure reporting system will be established.

Successively, secondary outcomes will be measured on the same individuals, from each cluster at discrete point in time using standardized and validated questionnaires and scales. The total length of the study trial will vary across arms. HCWs from arms with the longest trial duration will have four discrete outcome measurements taken, i.e., at baseline, during the last 2 weeks of P1, at 22 weeks after the intervention introduction, at the end of observation period 2 (P2) (first follow-up FU1), at 47 weeks at the end of P3 (FU2), and at 72 weeks from the intervention, at the end of P4 (FU3) (Fig. 1). These arms will have an overall trial duration of 87 weeks. Arms with shorter durations will have fewer outcome measurements taken. Data collection tools will be programmed electronically on tablets, using Open Data Kit (ODK) [51]. The questionnaire will be administered in French in DRC and in Arabic or English in Iraq and will include questions about HCWs exposure to violence, either to physical or verbal violence, the immediate actions taken if any after being exposed to violence, whether they reported the violence, or if any action was taken by the authorities. Questions will also inquire about family members’ or colleagues’ exposure to any sort of violence and the perspective of the participants about the future of this phenomenon. The questionnaire will also include validated scales to assess different psychosocial variables as for example, post-traumatic stress disorders (PTSD), the level of burnout, or measures of quality of life and cost data (see Table 3 Participant timeline).

**Table 3:** Sample size

	Iraq			DRC		
Drop out probability within an observation period	10%	20%	30%	10%	20%	30%
Expected number of health care workers to be recruited	340	415	491	212	259	307

### Plans to promote participant retention and complete follow-up {18b}

Plans to promote participant retention and complete follow-up will include maintaining effective communication with participants to ensure their understanding of the importance of the study and their role. The research study team will provide clear information about the study design, procedures, and potential benefits and risks to participants and will be in personalized contact with participants to help maintaining their engagement and interest in the study, through regular reminders and feedbacks on the progress of the study. Flexibility in scheduling interviews, follow-ups, and accommodating participants’ schedules will help reduce participant burden and increase their willingness to continue participation in the study.

### Data management {19}

All data collected will be hosted by the Swiss TPH. All data will be collected and stored in the form of pseudonyms. In addition, the data will be protected by cryptographic protocols. The secure encryption protocol (SSL protocol) will be used, capable of ensuring the security of communication on the Internet. As soon as the data is transferred to the server and a quality control has been carried out, no data will be stored on the local devices. The SOPs will serve as a guide for all employees on these principles.

### Confidentiality {27}

Data will be stored on a secure and password-protected server at Swiss TPH, Basel. The data will be collected on electronic tablets and will be uploaded on a daily basis in encrypted form. Project data will be handled with discretion and will only be accessible to authorized personnel. On data collection instruments and other project specific documents, participants are only identified by a unique participant number. In order to keep track of individual HCWs during the SW-RCT, each person enrolled will be assigned a code to allow retrieving the same participant at each follow-up step. Codes will only be revealed to the co-PIs in the field and to the research assistants assigned to data collection and destroyed immediately thereafter to protect the respondent and the interviewer. The list linking names and codes will be securely stored at Swiss TPH during the various data collection cycles and will be destroyed once the data has been linked. Only the PI and data analysts will have access to the source documents that link the individuals with the encoded data. Once the data has been encoded, access to the data will be permitted for monitoring, auditing, or data quality assessments.

### Plans for collection, laboratory evaluation, and storage of biological specimens for genetic or molecular analysis in this trial/future use {33}

N/A. The trial does not plan the collection, laboratory evaluation, or storage of biological samples for genetic or molecular analysis.

## Statistical methods

### Statistical methods for primary and secondary outcomes {20a}

Descriptive statistics will be used to summarize the characteristics of HCWs. For the analysis of observed event data, the model applied with the simulated data sets will again be used. HCWs will be considered as separate observational units. For each observation period, the time during which a HCW was under observation will be used as individual offset variable and each HCW will get his/her own random intercept in the analysis. Additional analyses will involve scores reported by the HCW at the end of each period or even during periods. Mixed linear models with robust standard error estimates involving analogous terms as the mixed Poisson regression models will be used; however, without accounting for the time, a HCW has been active during a given period. Analyses addressing the occurrence of severe events will be conducted using mixed logistic regression models. In this case, the outcome is 1 if the respective health worker experienced a severe event in the respective period and 0 otherwise. The structure of these models will be analogous to the one of the models for event rates and scores.

### Interim analyses {21b}

Analyses are done regularly to check data quality and provide feedback and advice to study teams, but no intermediate analyses of main endpoints are done.

### Methods for additional analyses (e.g., subgroup analyses) {20b}

Subgroup analyses will include exploring whether the intervention effects vary significantly between subcategories of trial participants, such as by age, gender, and year of experience in healthcare, by comparing the outcomes between the different groups. Various characteristics at the level of health care facility or HCWs (e.g., the severity of patient aggression or the location of the health care facility) might modify the intervention effects or also act as confounders if they are not optimally randomized. Stratified analyses and analyses involving interaction terms will be used to assess effect modification by the respective characteristics and to adjust for their potential confounding effect. Controlling for these factors in the statistical models will enable refined analysis. The analysis will also look if the intervention has a differential impact over time. For example, it is possible that the intervention’s effectiveness will improve as the HCWs become more familiar with the de-escalation techniques or as they have more opportunities to apply the skills learned in the training.

### Methods in analysis to handle protocol non-adherence and any statistical methods to handle missing data {20c}

Missing data due to protocol non-adherence or other reasons such as dropouts or missing outcome data will be handled statistically using methods such as multiple imputation and inverse probability weighting.

### Plans to give access to the full protocol, participant level-data, and statistical code {31c}

The full protocol and anonymized participant-level data will be made available upon request to the principal investigator.

## Oversight and monitoring

### Composition of the coordinating center and trial steering committee {5d}

The Swiss TPH will serve as the coordinating institution. The local co-principal investigators in both DRC and Iraq will be responsible to implement the trial and will be in direct contact with all of the participating study sites. The trial steering committee comprises the Principal Investigators of the study (representing Swiss TPH) and a representative from the ICRC. The trial steering committee will meet regularly, typically bi-weekly, and review the ongoing progress and conduct of the trial. The data management team is represented by the Swiss TPH and will conduct the statistical analysis.

### Composition of the data monitoring committee, its role, and reporting structure {21a}

A data monitoring committee (DMC) will not be required for the type of behavioral intervention that will be tested, as it will not involve testing drugs or other medical interventions that may pose significant risks to the participants.

### Adverse event reporting and harms {22}

The de-escalation violence training itself is not expected to pose any significant physical risks to the participants. However, there may be some physical and psychological risks associated with trial participation, especially if participants are required to confront potentially violent situations during the trial. Participants may experience stress, anxiety, or other negative emotional reactions as well physical injuries as a result of their participation. The trial will include measures to ensure the safety and well-being of the participants, as psychological support for affected HCWs.

### Frequency and plans for auditing trial conduct {23}

The oversight of the trial in each setting is managed by the Project Management Group, consisting of local researchers who convene bi-weekly to review trial conduct, ensuring protocol adherence and data quality.

A formal Trial Steering Group is not established; decision-making responsibilities are integrated within the Project Management Group. Given the low-risk nature of the intervention and the involvement of experienced local researchers, a separate Data Monitoring Committee was not formed.

### Plans for communicating important protocol amendments to relevant parties (e.g., trial participants, ethical committees) {25}

Any significant protocol amendment will be approved and submitted to Ethics Committees/IRBs prior to implementation and be notified to the appropriate parties in accordance to the local regulations.

### Dissemination plans {31a}

Trial results will be made available through publication in peer-reviewed indexed journals, conference presentations, and on the Swiss TPH, HCiD, and Elrha websites. Furthermore, the ICRC’s HCiD initiative has built a coalition of global stakeholders and will be uniquely placed to disseminate the research findings among humanitarians and health policy maker organizations via global, regional, and local forums.

## Discussion

Violence against health care is a global emergency, particularly in fragile contexts. Research on the patterns and motivations of violence in post-crisis settings from the Africa and Middle East regions is scarce. This study will adopt a stepped-wedge cluster randomized controlled trial design to investigate the impact of a contextualized de-escalation of violence training for health care workers (HCWs) and of a co-designed code of conduct for both clients and HCWs at the level of the health facilities, on the incidence and severity of both verbal and physical violent episodes in two post-conflict settings, the Democratic Republic of Congo (DRC) and Iraq. Strengthening social accountability by involving communities in researching causes of violence against HCWs and in designing solutions will enhance the appropriate understanding of cultural and social factors behind the motivations of violence in these settings, and most importantly, communities will become advocates for a sustainable intervention. Thus, the bottom-up crafting of rules for the code of conduct will enhance acceptability and sustainability of the intervention and will allow contextualizing the trainings on de-escalation of violence for HCWs. Following the participant longitudinally during the SW-RCT will yield a comprehensive understanding of the effect of the de-escalation violence training in isolation or in combination with the code of conduct on the incidence and severity of episodes of violence and whether the adapted de-escalation violence training would prevent violence in the early stages. At the same time, the strong research methodology will be essential in identifying the most (cost-) effective way to implement interventions in urban and rural areas in these contexts. Further, it is also expected that the intervention will have an impact on specific outcomes measured through the CCPAI and MBI score or the Physician Empathy scale, meaning that the adapted de-escalation training is expected to also contribute to increased confidence, less burnout, and increased empathy among HCWs.

It is expected that this research will contribute to developing a healthier and safer work environment for HCWs improving their psycho-physical well-being and adding to a general improvement in the quality of services. The approach taken in this study has been designed with the aim of facilitating the integration of the violence de-escalation training into professional training programs, as well as the development and implementation of hospital policies aimed at reducing violence.

## Trial status

The protocol version number is 1.0, dated November 11, 2021. Recruitment for the citizen science component began in April 2022. Participant’s recruitment to the SW-CRT began on November 2022. The target completion date is August 2023.

### Supplementary Information

**Additional file 1.**

## Data Availability

Access to the final trial dataset is granted to the trial sponsor, investigators, and other authorized parties such as ethics committees, upon request.

## Abbreviations

CAW: Community advocacy workshop
CS: Citizen scientist
DRC: Democratic Republic of Congo
ERSP-UCB: Ecole Régionale de Santé Publique, Université Catholique de Bukavu
FGD: Focus group discussion
HCiD: Health Care in Danger Initiative
HCW: Health care worker
ICRC: International Committee of the Red Cross
KII: Key informant interview
LMICs: Low– and middle-income countries
MBI: Maslach Burnout Inventory Scale
ODK: Open Data Kit
PTSD: Post traumatic stress disorder
PWG: Participatory working group
Swiss TPH: Swiss Tropical and Public Health Institute
SW-RCT: Stepped wedge randomized controlled trial
JSE-HP: Jefferson Scale of (Physician) Empathy
