# Supplementary material for: Understanding context of violence against healthcare through citizen science and evaluating the effectiveness of a co-designed code of conduct and of a tailored de-escalation of violence training in Eastern Democratic Republic of Congo and Iraq: a study protocol for a stepped wedge randomized controlled trial
Source: Trials. 2023 Dec 19;24:814. doi: 10.1186/s13063-023-07839-3 (PMC10729574; doi:10.1186/s13063-023-07839-3)
Supplement: Supplementary file 1 — Additional file 1. [file 13063_2023_7839_MOESM1_ESM.docx]

## Supplementary file 1

## Stepped-wedge cluster randomized study: calculate the statistical power

Power simulations were performed separately for the study in Iraq and the study in DRC. The assumptions underlying the simulations were:

1. The average number of events per period and health worker without any intervention (i.e., at baseline) equals 1.
2. The variation in the mean number of events across facilities was introduced by normally distributed terms with a mean of 0 and a standard deviation of 0.2 on the log-scale of expected events at the level of facilities.
3. The variation in the mean number of events across health workers within facilities was introduced by normally distributed terms with a mean of 0 and a standard deviation of 0.2 on the log-scale of expected events at the level of individual health workers.
4. The reduction in the event rate by intervention I compared to baseline in the first observation period following the intervention was assumed to be 40%, while the decrease was assumed to be 30% in period P3 in arms 1, 4 and 7 (i.e., in the second observation period after implementation of I).
5. The reduction in the event rate by intervention B compared to baseline was assumed to be 40% in all periods following the implementation of B.
6. The reduction in the event rate by intervention R compared to baseline was assumed to be 40%.
7. No period effect was assumed but time was included as covariate in the mixed Poisson regression model used to analyze the simulated data sets. The assumption of no time trend does not strongly influence power estimates provided that the true time trend is linear and constant across facilities and that the variable time is included in the model used to analyze the simulated data sets.
8. The probability of a health worker to leave the facility within a given observation period was assumed to be 10, 20 or 30%. For health workers quitting in a given period, the duration of observation was assumed to be half of the length of the respective study period, thus implying half the number of observable events in this case.
9. Simulations were conducted with and without replacement of health workers. If health workers were replaced, then their replacements were introduced only at the beginning of the next period. For health workers who left in a given period, no observations were generated in the following periods.
10. Effects of different interventions were assumed to be additive on the log scale of expected events.

Further assumptions were

1. The number of facilities per study arm equals 2 for Iraq and 3 for DRC
2. The number of health workers per facility equals 12 for Iraq and 5 for DRC.

The analysis of the simulated data sets was done using a mixed Poisson regression model with random intercepts for facilities and for health workers within facilities, with the fixed factors I (1 vs. 0), B (1 vs. 0) and R (1 vs. 0) and with an indicator variable for the third observation period in arms 1, 4 and 7 to capture the simulated decrease in the effect of I in P3 within these arms (cf. assumption d)). Moreover, time elapsed between the beginning of the study and the midpoint of the respective period was included as additional covariate. The natural logarithm of the length of observation of the respective health worker in the respective period was used as offset variable in the model. As results of the two studies can be combined using weights proportional to the inverse standard errors squared, we also estimated the power of obtaining statistically significant summary estimates.
